# Metabolic Syndrome Is Associated With Impaired Insulin-Stimulated Myocardial Glucose Metabolic Rate in Individuals With Type 2 Diabetes: A Cardiac Dynamic ^18^F-FDG-PET Study

**DOI:** 10.3389/fcvm.2022.924787

**Published:** 2022-06-29

**Authors:** Elena Succurro, Patrizia Vizza, Annalisa Papa, Francesco Cicone, Giuseppe Monea, Giuseppe Tradigo, Teresa Vanessa Fiorentino, Maria Perticone, Pietro Hiram Guzzi, Angela Sciacqua, Francesco Andreozzi, Pierangelo Veltri, Giuseppe Lucio Cascini, Giorgio Sesti

**Affiliations:** ^1^Department of Medical and Surgical Sciences, University Magna Graecia of Catanzaro, Catanzaro, Italy; ^2^Research Center for the Prevention and Treatment of Metabolic Diseases (CR METDIS), University Magna Graecia of Catanzaro, Catanzaro, Italy; ^3^Department of Experimental and Clinical Medicine, Magna Graecia University of Catanzaro, Catanzaro, Italy; ^4^Ecampus University, Novedrate, Italy; ^5^Department of Clinical and Molecular Medicine, University of Rome-Sapienza, Rome, Italy

**Keywords:** myocardial glucose metabolism, metabolic syndrome, type 2 diabetes, cardiovascular disease, insulin resistance, cardiac ^18^F-FDG-PET

## Abstract

Metabolic syndrome is a condition characterized by a clustering of metabolic abnormalities associated with an increased risk of type 2 diabetes and cardiovascular disease. An impaired insulin-stimulated myocardial glucose metabolism has been shown to be a risk factor for the development of cardiovascular disease in patients with type 2 diabetes. Whether cardiac insulin resistance occurs in subjects with metabolic syndrome remains uncertain. To investigate this issue, we evaluated myocardial glucose metabolic rate using cardiac dynamic ^18^F-FDG-PET combined with euglycemic-hyperinsulinemic clamp in three groups: a group of normal glucose tolerant individuals without metabolic syndrome (*n* = 10), a group of individuals with type 2 diabetes and metabolic syndrome (*n* = 19), and a group of subjects with type 2 diabetes without metabolic syndrome (*n* = 6). After adjusting for age and gender, individuals with type 2 diabetes and metabolic syndrome exhibited a significant reduction in insulin-stimulated myocardial glucose metabolic rate (10.5 ± 9.04 μmol/min/100 g) as compared with both control subjects (32.9 ± 9.7 μmol/min/100 g; *P* < 0.0001) and subjects with type 2 diabetes without metabolic syndrome (25.15 ± 4.92 μmol/min/100 g; *P* = 0.01). Conversely, as compared with control subjects (13.01 ± 8.53 mg/min x Kg FFM), both diabetic individuals with metabolic syndrome (3.06 ± 1.7 mg/min × Kg FFM, *P* = 0.008) and those without metabolic syndrome (2.91 ± 1.54 mg/min × Kg FFM, *P* = 0.01) exhibited a significant reduction in whole-body insulin-stimulated glucose disposal, while no difference was observed between the 2 groups of subjects with type 2 diabetes with or without metabolic syndrome. Univariate correlations showed that myocardial glucose metabolism was positively correlated with insulin-stimulated glucose disposal (*r* = 0.488, *P* = 0.003), and negatively correlated with the presence of metabolic syndrome (*r* = −0.743, *P* < 0.0001) and with its individual components. In conclusion, our data suggest that an impaired myocardial glucose metabolism may represent an early cardio-metabolic defect in individuals with the coexistence of type 2 diabetes and metabolic syndrome, regardless of whole-body insulin resistance.

## Introduction

Metabolic syndrome (MetS) is a condition characterized by a clustering of metabolic abnormalities, including abdominal obesity, dyslipidemia, impaired glucose tolerance, and/or hypertension, and is associated with an increased risk for type 2 diabetes (T2DM) and cardiovascular morbidity and mortality (1–6). Each component of the MetS is an independent cardiovascular risk factors and several studies have reported that the combination of these risk factors increases the severity of a spectrum of cardiovascular alterations including microvascular dysfunction, coronary atherosclerosis, cardiac dysfunction, and heart failure (2–7). Data from the Botnia Study reported a prevalence of MetS ranging from 78 to 84% in subjects with T2DM (4). The coexistence of MetS and T2DM is associated with more than double the risk of coronary heart disease (CHD) compared to other combinations of MetS risk factors (6). Moreover, large-scale prospective studies have shown that MetS is predictive of increased CHD mortality in patients with T2DM (4, 6, 7). Insulin resistance is considered central to the pathophysiology of MetS and seems to be a key element for the development of atherosclerotic process responsible of its increased CV risk (1, 7). However, the underlying pathophysiologic mechanisms of this complex disorder remain incompletely understood.

Prior studies have shown an impaired insulin-stimulated myocardial glucose metabolism in patients with type 2 diabetes with or without CHD and in subjects at increased risk of T2DM (8–11). A decrease of myocardial glucose uptake was also observed in patients with prediabetes or T2DM and heart failure compared to normoglycemic individuals (12). Furthermore, an inverse association between myocardial glucose uptake and coronary atherosclerosis has been reported (13).

A direct relationship has been demonstrated between impaired insulin-stimulated myocardial glucose uptake and reduced peripheral insulin sensitivity in patients with T2DM and in individuals with prediabetes (9, 14). However, the extent to which cardiac insulin resistance is due to T2DM *per se* vs. metabolic syndrome is still unsettled. To this aim, we evaluated insulin-stimulated myocardial glucose metabolism using cardiac dynamic Myocardial positron emission tomography (PET) with ^18^F-Fluorodeoxyglucose (^18^F-FDG) combined with euglycemic-hyperinsulinemic clamp in T2DM individuals with or without MetS.

## Materials and Methods

### Study Participants

The study cohort comprised 35 subjects participating in the CATAnzaro MEtabolic RIsk factors (CATAMERI), an ongoing observational study recruiting adult individuals with one or more cardio-metabolic risk factors recruited at a referral university hospital of the University “Magna Graecia” of Catanzaro (9, 15). Eligible subjects were recruited according to the following inclusion criteria: age between 30 and 70 years, and positivity for one or more cardio-metabolic risk factors including family history of diabetes, dysglycemia, hypertension, dyslipidemia, and overweight/obesity. Exclusion criteria were type 1 diabetes, end-stage renal disease, previous cardiovascular disease on the basis of medical history and resting electrocardiogram, liver cirrhosis, history of malignant or autoimmune diseases, acute or chronic infections, medicaments affecting heart function including beta blockers and, for non-diabetic subjects, treatment with drugs known to influence glucose tolerance such as steroids and estro-progestins. All subjects underwent anthropometrical evaluation including measurements of body mass index (BMI), and waist circumference and body composition by bioelectrical impedance, blood pressure, and biochemical determinations. After an overnight fasting, a 75 g OGTT was performed in individuals with FPG < 126 mg/dl, HbA1c < 6.5% and no history of T2DM. Glucose tolerance status was defined on the basis of OGTT according to the American Diabetes Association (ADA) criteria (16). According to the ADA recommendations (16), subjects with dysglycemia were classified as prediabetes when either fasting plasma glucose (FPG) was 100–125 mg/dl (5.5–6.9 mmol/l) or 2-h post-load plasma glucose value during a 75-g OGTT was 140–199 mg/dl (7.77–11.0 mmol/l) or HbA1c 5.7–6.4%, type 2 diabetes when either FPG was ≥126 mg/dl (>7 mmol/l), or 2-h post-load glucose ≥200 mg/dl (>11.1 mmol/l), or HbA1c ≥6.5% or were taking treatment with hypoglycemic agents. Individuals were classified as having normal glucose tolerance (NGT) when FPG was <100 mg/dl (5.5 mmol/l), 2-h post-load glucose <140 mg/dl (<7.77 mmol/l) and HbA1c <5.7% (16). Patients with T2DM were divided in two groups according to the presence or absence of MetS defined according to American Heart Association and National Heart, Lung, and Blood Institute criteria (17). MetS is defined by the presence of three of these factors: (a) waist circumference ≥ 102 cm for men and ≥ 88 cm for women; (b) TG increased plasma levels [>150 mg/dL (1.7 mmol/L)] or specific treatment with lipid-lowering drugs; (c) reduced high-density lipoprotein (HDL)-cholesterol [ <40 mg/dL (1.0 mmol/L) in men; <50 mg/dL (1.3 mmol/L) in women] or specific treatment with lipid-lowering drugs; (d) increased blood pressure (systolic ≥ 130 mm Hg and/or diastolic ≥ 85 mm Hg) or treatment of previously diagnosed hypertension; and (e) increased FPG levels [>100 mg/ dL (5.6 mmol/L)] or treatment of T2DM.

The study was approved by the Hospital ethical committee (Comitato Etico Azienda Ospedaliera “Mater Domini”) and all participants provided their written informed consent to participate in this study in accordance with principles of Helsinki Declaration.

### ^18^F-FDG PET Scan Combined With Euglycemic Hyperinsulinemic Clamp

Myocardial metabolic rate of glucose was measured by ^18^F-FDG-PET acquired in the course of euglycemic hyperinsulinemic clamp as previously described (9). Subjects received a priming dose of insulin (Humulin R 100 UI/ml; Eli Lilly) during the initial 10 min to acutely raise the desired levels of plasma insulin, followed by continuous insulin infusion fixed at 40 mU/m^2^ × min. The blood glucose level was maintained constant at 90 mg/dl for the next 120 min by infusing 20% glucose at varying rates according to blood glucose measurements performed at 5-min intervals (mean coefficient of variation of blood glucose was <4%). Glucose metabolized by the whole body (M) was calculated as the mean rate of glucose infusion measured during the last 60 min of the clamp examination (steady state) and was expressed as milligrams per minute per kilogram fat-free mass (M_FFM_).

The ^18^F-FDG PET imaging procedure was performed on a hybrid PET/CT scanner (GE Discovery ST8- 2D PET scanner), starting 60 min after the insulin infusion. Patients were positioned supine under the camera, and the correct collimation to the chest was checked by a scout CT image. 60-min dynamic acquisition was started simultaneously with the intravenous injection of 370 MBq18F-FDG. A continue frame mode acquisition was performed as follow: 8 × 15 s, 2 × 30 s, 2 × 120 s, 1 × 180 s, 6 × 300 s, 1 × 600 s (18). After a 10-min break, a late time-point static image of 1,200 s was acquired (i.e., between 70 and 90 min post-injection). PET images were corrected for decay and attenuation, then reconstructed in a 128 X 128 matrix using a OSEM algorithm Decay- corrected images were reconstructed in a 128 X 128 matrix using a OSEM algorithm and CT-based attenuation correction was performed. The insulin-glucose infusion continued during entire PET acquisition. The estimation of myocardial MRGlu was performed using a Patlak compartmental modeling (9, 19), a widely diffuse technique provided by a graphical tool specific for cardiac images analysis (PCARD) in PMOD Software platform (Version 3.806) (19). PCARD allows to measure the global MRGlu as well as the segmental myocardial glucose rate by using a segmentation algorithm to divide myocardium into standard 17 segments model according to American Society of Nuclear Cardiology guideline and the American Heart Association (20). Segments are then grouped in three vascular territories corresponding to the left anterior descending (LAD) artery, left circumflex (LCX) artery, and right coronary artery (RCA).

### Laboratory Determinations

Plasma glucose, total and HDL cholesterol and triglycerides were measured by enzymatic methods (Roche Diagnostics, Mannheim, Germany). HbA1c was measured with high performance liquid chromatography using an NGSP-certified automated analyzer (Adams HA-8160 HbA1c analyzer, Menarini, Italy). Plasma insulin concentration was measured with a chemiluminescence-based assay (Immulite, Siemens, Italy).

### Statistical Analyses

Variables with skewed distribution, such as triglycerides and MFFM, were natural log transformed for statistical analyses. Continuous variables are expressed as means ± SD. Categorical variables were compared by chi-square test (χ2). Comparisons between study groups were performed using a general linear model (GLM) with *post-hoc* Fisher's least significant difference correction for pairwise comparisons, utilized for comparisons which reached a statistical significant *p*-value in the GLM. Relationships between variables were determined by Pearson's correlation coefficient (*r*). Stepwise multivariate regression analysis was run to determine the independent predictors of myocardial glucose metabolism.

Considering that previous studies have reported a reduction of 41–50% of myocardial glucose uptake in subjects with type 2 diabetes or prediabetes (8–10), we calculated that five subjects for each group had 80% power to detect a 50% difference in myocardial glucose uptake with an alpha of 0.05. With an addition of 15% to safeguard from potential missing values, a sample size of at least six subjects for each group was planned.

For all analyses, a *P*-value ≤ 0.05 was considered to be statistically significant. All analyses were performed using SPSS software Version 22 for Windows.

## Results

### Clinical Characteristics and Metabolic Parameters of the Study Subjects

The present study includes 35 individuals: 25 subjects had T2DM and 10 were normal glucose tolerant individuals without MetS. Subjects with T2DM were divided into two subgroups according to the presence of MetS: 19 subjects with T2DM and MetS (T2DM MetS), and 6 subjects with T2DM without MetS [T2DM Non-MetS (*n* = 6)]. Anthropometric and biochemical characteristics of the study participants are shown in Table 1. All the subjects with type 2 diabetes were treated with metformin. As compared to control subjects, T2DM MetS and Non-Mets subjects were older and more frequently female. Therefore, all the analyses resulted significant were then adjusted for age and gender.

**Table 1:** Differences in clinical characteristics of subjects with T2DM according to metabolic syndrome compared to control subjects.

	**Control group** **(*n* = 10)**	**T2DM**		**Control vs. T2DM non-MetS**		**Control vs. T2DM MetS**		**T2DM Non-MetS vs. T2DM MetS**	
		**Non-MetS** **(*n* = 6)**	**MetS** **(*n* = 19)**	***P*-value**	***P*-value^**§**^**	***P*-value**	***P*-value^**§**^**	***P*-value**	***P*-value^**§**^**
Gender (M/F)	7/3	1/5	7/12	0.005	0.005	0.06	–	0.09	–
Age (yrs)	42.4 ± 9	57.8 ± 11	55.1 ± 7	0.002	0.002	0.001	0.001	0.5	–
BMI (kg/m^2^)	27.4 ± 5.09	29.4 ± 4.8	31.8 ± 4.2	0.2	–	0.01	0.009	0.5	–
Waist circumference (cm)	95.8 ± 12	104 ± 8	109 ± 9	0.7	–	0.003	0.01	0.2	–
Systolic blood pressure (mmHg)	110 ± 15	117 ± 13	130 ± 11	0.7	–	<0.0001	0.01	0.03	0.02
Diastolic blood pressure (mmHg)	71 ± 8	74 ± 11	79.9 ± 10	0.5	–	0.04	0.2	0.08	–
Total cholesterol (mg/dl)	178 ± 32	164 ± 24	184 ± 41	0.4	–	0.7	–	0.06	–
HDL (mg/dl)	57.3 ± 11	41.5 ± 9	43.9 ± 8	0.003	0.3	0.001	0.07	0.7	–
Triglycerides (mg/dl)	88 ± 44	98 ± 42	160 ± 73	0.7	–	0.004	0.01	0.04	0.1
Fasting glucose (mgl/dL)	86 ± 5	117 ± 14	149 ± 41	0.02	0.002	<0.0001	0.001	0.02	0.1
2-h post load plasma glucose (mg/dl)	111 ± 11	–	–	–	–	–	–	–	–
HbA1c (%)	5.2 ± 0.5	7.1 ± 0.8	7.6 ± 1.1	0.001	0.003	<0.0001	<0.0001	0.8	0.8
Fasting plasma insulin (mU/mL)	11.4 ± 6.3	9.3 ± 7.4	14.8 ± 7.6	0.5	–	0.2	–	0.1	–
Diabetes duration (yrs)	–	1.2 ± 2.5	6.9 ± 5.6	–	–	–	–	0.1	0.1
Antihypertensive therapy (%)	10	50	73.7	0.006	0.6	<0.0001	0.06	0.1	0.1
Lipid-lowering therapy (%)	0	16.7	68.4	0.5	–	<0.0001	0.005	0.008	0.04
**Glucose-lowering therapy**									
Metformin (%)	-	100	100	–	–	–	–	1	1
**Number of risk factors of metabolic syndrome (** ***n*** **)**									
0	5	0	0	0.03	0.06	<0.0001	<0.0001	<0.0001	0.001
1 or 2	5	6	0						
3 ore more	0	0	19						

*Continous data are expressed as means ± SD. Comparisons between the groups were performed using a general linear model. Categoric variables were compared by χ2 test. BMI, Body Mass Index; HDL, High Density Lipoprotein; FPG, Fasting Plasma Glucose. ^§^P-values refer to results after analyses with adjustment for age and gender*.

As expected, after adjustment for age and gender, T2DM MetS and T2DM Non-MetS individuals had significantly higher fasting plasma glucose (FPG) and HbA1c as compared with control subjects. On the contrary, no significant differences were observed in fasting insulin levels between the three groups in study. In addition, as compared with non-diabetic subjects, T2DM MetS individuals exhibited significantly higher BMI (27.4 ± 5.09 vs. 31.8 ± 4.2 kg/m^2^, *P* = 0.009), waist circumference (95.8 ± 12 vs. 109 ± 9 cm, *P* = 0.01), systolic blood pressure (sBP) (110 ± 15 vs. 130 ± 11 mmHg, *P* = 0.01) and triglycerides levels (88 ± 44 vs. 160 ± 73 mg/dl, *P* = 0.01). Moreover, as compared with control subjects, a higher proportion of T2DM MetS individuals were treated with lipid-lowering therapies and exhibited a higher number of risk factors of metabolic syndrome. No differences were observed in diastolic blood pressure (dBP), total and HDL cholesterol and antihypertensive treatment (Table 1).

After adjustment for age and gender, no significant differences were observed between T2DM Non-MetS individuals and control subjects in BMI (29.4 ± 4.8 vs. 27.4 ± 5.09 kg/m^2^, *P* = 0.2) and waist circumference (104 ± 8 vs. 95.8 ± 12 cm, *P* = 0.7) (Table 1). Furthermore, no significant differences between T2DM Non-MetS individuals and control subjects were found with respect to blood pressure, lipid profile and antihypertensive and lipid-lowering therapies (Table 1).

After adjustment for age and gender, no significant differences between T2DM with and without MetS subjects were found with respect to BMI (31.8 ± 4.2 vs. 29.4 ± 4.8 kg/m^2^, *P* = 0.5) and waist circumference (109 ± 9 vs. 104 ± 8 cm, *P* = 0.2) (Table 1). In addition, no significant differences between T2DM groups were found with respect to glycemic parameters, lipid profile, diabetes duration, antihypertensive and glucose-lowering therapy (Table 1). T2DM MetS subjects exhibited significantly higher sBP and a higher number of risk factors of metabolic syndrome and a higher proportion were treated with lipid-lowering therapies as compared with T2DM Non-MetS (Table 1).

### Myocardial Glucose Metabolic Rate and Insulin Sensitivity in the Study Subjects

As compared with control subjects (13.01 ± 8.53 mg/min × Kg FFM), both T2DM MetS (3.06 ± 1.7 mg/min × Kg FFM, *P* = 0.008) and T2DM Non-MetS (2.91 ± 1.54 mg/min × Kg FFM, *P* = 0.01) exhibited a significant reduction in insulin-stimulated glucose disposal after adjusting for age and gender (Table 2).

**Table 2:** Differences in myocardial glucose metabolic rate and insulin sensitivity of subjects with T2DM according to metabolic syndrome compared to control subjects.

	**Control group** **(*n* = 10)**	**T2DM**		**Control vs. T2DM non-MetS**		**Control vs. T2DM MetS**		**T2DM Non-MetS vs. T2DM MetS**	
		**Non-MetS** **(*n* = 6)**	**MetS** **(*n* = 19)**	***P*-value**	***P*-value^**§**^**	***P*-value**	***P*-value^**§**^**	***P*-value**	***P*-value^**§**^**
Insulin-stimulated glucose disposal (mg/min x Kg FFM)	13.01 ± 8.53	2.91 ± 1.54	3.06 ± 1.7	<0.0001	0.01	<0.0001	0.008	0.6	–
Myocardial MRGlu (μmol/min/100 g)	32.9 ± 9.7	25.15 ± 4.92	10.5 ± 9.04	0.04	0.03	<0.0001	<0.0001	0.001	0.01
LAD Myocardial MRGlu (μmol/min/100 g)	32.41 ± 8.79	23.2 ± 5.6	10.03 ± 9.6	0.04	0.02	<0.0001	<0.0001	0.003	0.04
RCA Myocardial MRGlu (μmol/min/100 g)	33.69 ± 12.38	26.59 ± 4.7	9.9 ± 8.00	0.05	0.04	<0.0001	<0.0001	0.002	0.001
LCX Myocardial MRGlu (μmol/min/100 g)	32.79 ± 9.1	26.3 ± 5.7	11.7 ± 9.6	0.05	0.04	<0.0001	<0.0001	0.002	0.02

*Continous data are expressed as means ± SD. Comparisons between the groups were performed using a general linear model. MRGlu, glucose metabolic rate; LAD, left anterior descending; LCX, left circumflex; RCA, right coronary artery. ^§^P-values refer to results after analyses with adjustment for age and gender*.

T2DM MetS individuals exhibited a significant age and gender-adjusted reduction in myocardial glucose metabolic rate as compared with control subjects (10.5 ± 9.04 vs. 32.9 ± 9.7 μmol/min/100 g; *P* < 0.0001) (Table 2). Likewise, T2DM MetS individuals exhibited a significant reduction in myocardial MRGlu corresponding to the vascular territories sprayed by the LAD artery (*P* < 0.0001), RCA (*P* < 0.0001), and LCX artery (*P* < 0.0001) as compared with control subjects.

As compared with control subjects, T2DM Non-MetS individuals exhibited a significant age and gender-adjusted reduction in myocardial glucose metabolic rate (25.15 ± 4.92 vs. 32.9 ± 9.7 μmol/min/100 g; *P* = 0.03) (Table 2), and myocardial MRGlu corresponding to the vascular territories supplied by the LAD artery (*P* = 0.02), RCA (*P* = 0.04), and LCX artery (*P* = 0.04) (Table 2).

As compared with T2DM Non MetS subjects, T2DM MetS individuals exhibited a significant age and gender-adjusted reduction in myocardial glucose metabolic rate (10.5 ± 9.04 vs. 25.15 ± 4.92 μmol/min/100 g; *P* = 0.01) (Table 2). This difference remained significant also after adjustment for systolic blood pressure (*P* = 0.005). In addition, T2DM MetS individuals showed a significant decrease in myocardial MRGlu corresponding to the vascular territories supplied by the LAD artery (*P* = 0.04), RCA (*P* = 0.001), and LCX artery (*P* = 0.02) as compared with T2DM Non MetS subjects (Table 2).

No significant difference was observed in insulin-stimulated glucose disposal between T2DM subjects with and without MetS (*P* = 0.6) (Table 2).

### Association Between Metabolic Syndrome and Myocardial Glucose Metabolic Rate

**Figure 1:**
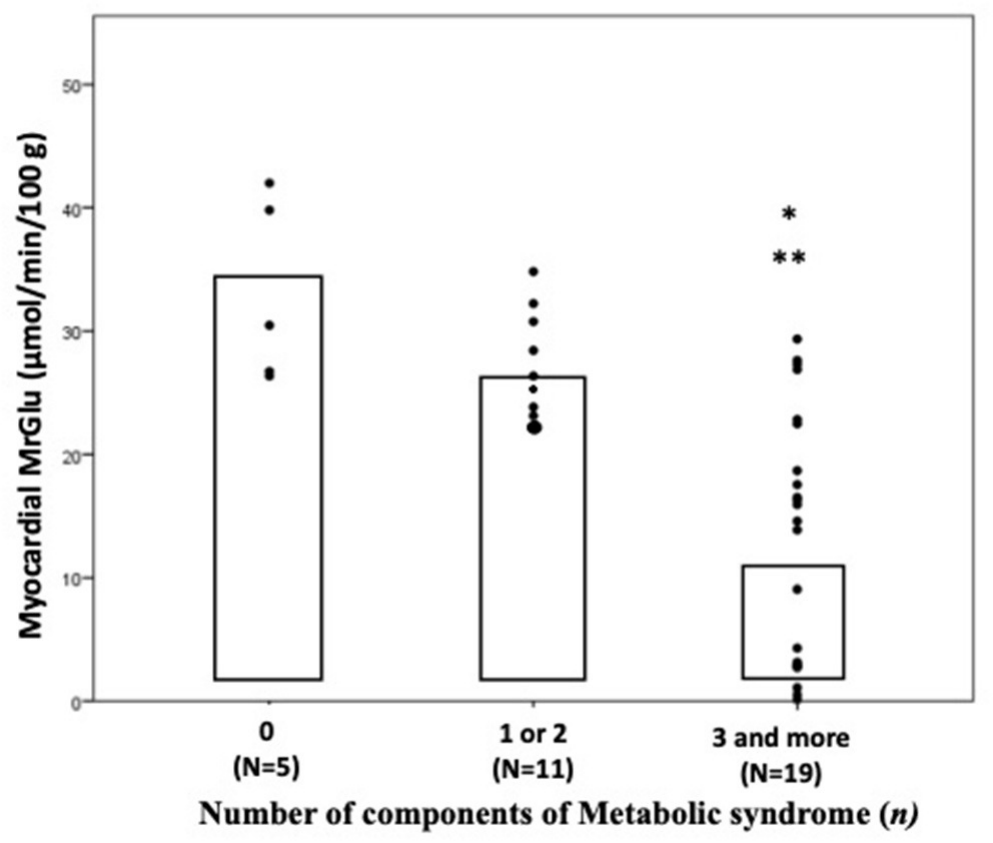
Myocardial glucose metabolic rate in subjects divided by number of components of metabolic syndrome. *P*-values refer to analyses after adjustment for age and sex. *P < 0.0001 vs. 0 component group; **P < 0.0001 vs. 1 or 2 components group. MrGlu, glucose metabolic rate.

As shown in Figure 1, after adjustment for age and gender, myocardial glucose metabolic rate progressively decreased in parallel with the increase of the number of MetS components. Notably, after adjustment for age and gender, subjects with 3 and more risk factors of MetS showed a significant decrease in myocardial MRGlu both than subjects with 1 or 2 risk factors (*P* < 0.0001) and those with none risk factors of MetS (*P* < 0.0001) (Figure 1).

**Figure 2:**
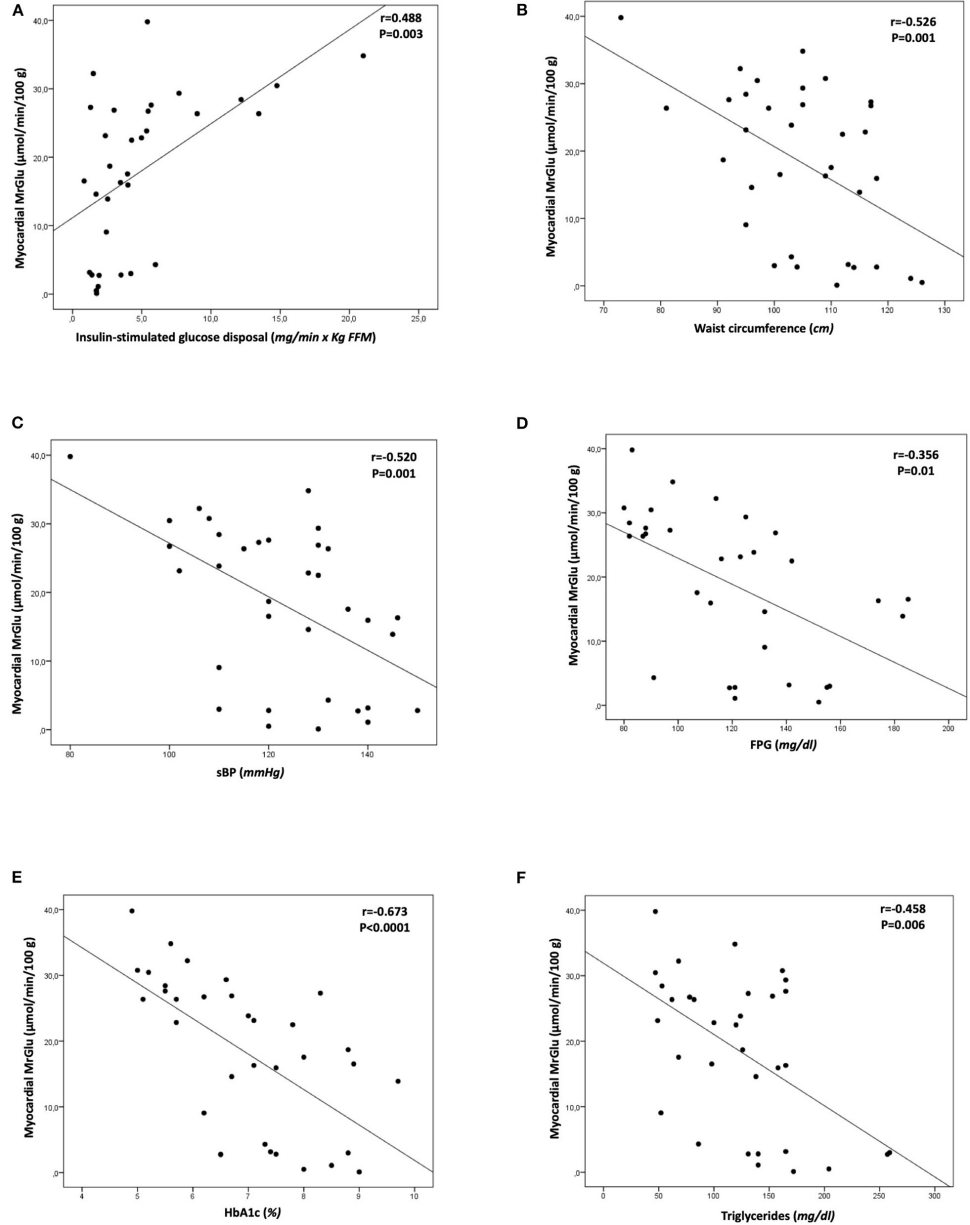
Univariate correlations between myocardial glucose metabolic rate and insulin-stimulated glucose disposal **(A)**, waist circumference **(B)**, systolic blood pressure **(C)**, fasting plasma glucose **(D)**, HbA1c **(E)**, triglycerides **(F)**. MrGlu, glucose metabolic rate; sBP, systolic blood pressure; FPG, fasting plasma glucose.

Univariate correlations showed that myocardial glucose metabolism was negatively correlated with the presence of MetS (*r* = −0.743, *P* < 0.0001), waist circumference (*r* = −0.526; *P* =0.001), sBP (*r* = −0.520; *P* = 0.001), FPG (*r* = −0.356; *P* = 0.01), HbA1c (*r* = −0.673; *P* < 0.0001), triglycerides (*r* = −0.458; *P* = 0.006), and positively correlated with insulin-stimulated glucose disposal (*r* = 0.488, *P* = 0.003) (Figure 2). No significant correlation was found between myocardial glucose metabolism and diabetes duration (*r* = −0.357, *P* = 0.1).

### Independent Predictors of Myocardial Glucose Metabolic Rate

To evaluate the independent contributors to myocardial glucose metabolism, we performed a stepwise multivariate regression analysis running a model including age, gender, BMI, waist circumference, blood pressure, lipid profile, fasting plasma glucose, HbA1c, fasting plasma insulin, insulin-stimulated glucose disposal, diabetes duration, presence of metabolic syndrome. The variables that remained significantly associated with myocardial MRGlu were presence of metabolic syndrome (β = −0.625; *P* = 0.002) and insulin-stimulated glucose disposal (β = 0.463; *P* = 0.01) explaining 55.9% of its variation (Table 3).

**Table 3:** Independent predictors of myocardial glucose metabolism after stepwise multiple regression analysis.

	***Partial r*** **^2^ (%)**	***Total r*** **^2^ (%)**	**β**	***P***
Presence of Metabolic Syndrome	34.5	34.5	−0.625	0.002
Insulin-stimulated glucose disposal (mg/min x Kg FFM)	21.3	55.9	0.463	0.01

*Model including age, gender, BMI, waist circumference, blood pressure, lipid profile, fasting plasma glucose, HbA1c, fasting plasma insulin, insulin-stimulated glucose disposal, diabetes duration, presence of Metabolic Syndrome*.

## Discussion

The main finding of the present study is that subjects with type 2 diabetes and metabolic syndrome without coronary heart disease exhibit a greater degree of myocardial insulin resistance evaluated using the gold standard cardiac dynamic PET combined with euglycemic-hyperinsulinemic clamp technique as compared with diabetic subjects without metabolic syndrome. Notably, individuals with coexistence of T2DM and metabolic syndrome exhibited a reduction of 59% in insulin-mediated myocardial glucose metabolism than subjects with T2DM without MetS and a reduction of 68% as compared with control subjects. These differences in myocardial glucose metabolism between T2DM MetS and T2DM without MetS groups remained significant also after adjustment for systolic blood pressure (*P* = 0.005) thus arguing against the possibility that reduction in systolic blood pressure have a major role in cardiac insulin resistance. Previous studies have reported a reduction ranging between 22 and 41% in myocardial glucose uptake in patients with type 2 diabetes with or without coronary artery disease as compared with non-diabetic individuals (8, 10, 21, 22), but the impact of metabolic syndrome on myocardial glucose metabolism in patients with T2DM has not be evaluated. Our data are consistent with preclinical studies showing an impaired insulin-induced glucose uptake in MetS cardiomyocytes (23, 24). Myocardial glucose metabolic rate was positively correlated with whole-body insulin-stimulated glucose disposal (*r* = 0.488, *P* = 0.003) in keeping with several prior studies showing a direct relationship between whole-body insulin sensitivity and cardiac glucose metabolism in both non-diabetic and prediabetic and T2DM individuals (8–11). By contrast, two previous studies did not find a significant correlation between systemic insulin sensitivity and myocardial glucose uptake (25, 26). Possible explanations for this disparity include differences in the methods used to assess myocardial glucose uptake [differences in glucose and insulin infusion protocols, i.e., supraphysiological insulinization (200 mU × min^−1^ × m^2^ insulin clamp) after an overnight insulin infusion in T2DM], and the population analyzed (T2DM with ischemic heart failure vs. individuals without cardiovascular disease or heart failure in our study). Furthermore, for the first time, we found that myocardial glucose metabolic rate was negatively correlated with the presence of metabolic syndrome (*r* = −0.743, *P* < 0.0001) and with the individual factors, including waist circumference (*r* = −0.526; *P* =0.001), sBP (*r* = −0.520; *P* = 0.001), FPG (*r* = −0.356; *P* = 0.01) and triglycerides (*r* = −0.458; *P* = 0.006). Notably, the presence of metabolic syndrome was the major independent contributor of the impairment in myocardial glucose metabolism explaining 34.5% of its variation. Whole-body insulin resistance independently contributed by an additional 21.3% to impairment of cardiac glucose metabolism thus suggesting a direct effects of the components of the metabolic syndrome on cardiac insulin resistance. It is noteworthy that no differences in whole-body insulin-stimulated glucose disposal or in glycemic control and abdominal adiposity were observed between T2DM individuals with or without MetS indicating that worsening of cardiac insulin resistance is a feature **t**hat is exacerbated by the presence of metabolic syndrome. Moreover, to explore a potential relationship between diabetes duration and impaired myocardial glucose metabolism we performed a univariate analysis showing there was not a significant correlation. The hypothesis of the major role of metabolic syndrome in determining cardiac insulin resistance is further supported by the finding that myocardial glucose uptake progressively decreased with the increase of the number of components of metabolic syndrome. Subjects with 3 and more risk factors of MetS showed a 69% reduction in myocardial insulin-stimulated glucose metabolism than subjects with no risk factors, and a 56.7% reduction than those with 1 or 2 components of the metabolic syndrome. Overall, the results of the present study suggest that the risk factors clustering in the metabolic syndrome acting independently of impairment of whole-body insulin sensitivity can aggravate myocardial insulin resistance in subjects with type 2 diabetes without cardiovascular disease.

Large prospective studies have reported that the presence of metabolic syndrome in subjects with T2DM is associated with an increased CHD morbidity and mortality (4, 6, 7). In the Finnish study, metabolic syndrome predicted cardiovascular mortality in T2DM patients in earlier phases, before development of CV events (5). Our finding suggest that metabolic syndrome increases the impairment in insulin-stimulated myocardial metabolic rate of glucose in T2DM patients, and therefore, understanding pathological mechanism underlying the increased ischemic risk in subjects with MetS and adopting therapeutic strategies aimed to reduce the CV events may help improve outcomes in these patients.

In normal subjects, under physiological conditions the heart can use both free fatty acids (FFA) and glucose as energy substrates, thus providing metabolic flexibility. During fasting, FFA are the main fuel for myocardial oxidative metabolism, and glucose metabolism is relatively low. Under postprandial conditions, increase of insulin levels led to an increase in myocardial glucose utilization. Subjects with insulin resistance and individuals with T2DM have an impaired metabolic flexibility due to a reduction of GLUT-4 activity resulting in a reduction of myocardial glucose uptake and an increase of FFA oxidation, even in presence of hyperinsulinemia during the post-prandial state. These changes lead to mitochondrial dysfunction with a low energy production and, consequently, death of cardiomyocytes, cardiac dysfunction and therefore contribute to coronary heart disease (8, 12, 13, 27–29).

The present study has several strengths. To the best of our knowledge, this is the first study assessing in individuals with T2DM and metabolic syndrome insulin-stimulated myocardial and whole-body glucose metabolism using cardiac dynamic PET in combination with the euglycemic-hyperinsulinemic clamp technique, considered the gold standard technique because it allows the valuation of myocardial glucose uptake under uniform experimental conditions of euglycemia and physiological hyperinsulinemia thus removing the confounding factor of different circulating glucose and insulin levels (8, 14, 30). Moreover, we analyzed not only the global MRGlu but also the segmental myocardial glucose metabolism corresponding to the vascular territories of the major coronary arteries. Additionally, all tests including ^18^F-FGD PET scan combined with euglycemic hyperinsulinemic clamp were collected at the same time by a skilled staff after a standardized training.

The current study also has some limitations. The results are only based on Caucasian individuals aging between 30 and 70 years thus limiting the generalizability of the present results to other ethnicities or to younger and older individuals. Additionally, the cross-sectional design of the study precludes causal inferences. Furthermore, we did not measure FFA levels, and we have no data for myocardial glucose metabolism under basal condition thus precluding us to determine the potential metabolic flexibility of the groups in study. Finally, although statistical analyses were adjusted for a wide variety of covariates, residual confounders such as physical activity, and nutritional status, may have affected the results.

## Data Availability Statement

The original contributions presented in the study are included in the article/supplementary material, further inquiries can be directed to the corresponding author/s.

## Ethics Statement

The studies involving human participants were reviewed and approved by Hospital Ethical Committee (Comitato Etico Azienda Ospedaliera Mater Domini) Catanzaro, Italy. The patients/participants provided their written informed consent to participate in this study.

## Author Contributions

ES conceived the study, researched and analyzed data, and wrote and edited the manuscript. PVi analyzed the data from the cardiac PET scans. AP and FC performed cardiac PET scans. GM, GT, TVF, MP, PHG, and AS researched data and reviewed the manuscript. FA, PVe, and GC contributed to study design and to the discussion and reviewed the manuscript. GS conceived the study, analyzed data, wrote, reviewed, and edited the manuscript, is the guarantor of this work and, as such, had full access to all the data in the study and takes responsibility for the integrity of the data, and the accuracy of the data analysis. All authors have read and approved the final manuscript.

## Conflict of Interest

The authors declare that the research was conducted in the absence of any commercial or financial relationships that could be construed as a potential conflict of interest.

## Publisher's Note

All claims expressed in this article are solely those of the authors and do not necessarily represent those of their affiliated organizations, or those of the publisher, the editors and the reviewers. Any product that may be evaluated in this article, or claim that may be made by its manufacturer, is not guaranteed or endorsed by the publisher.
